# Effects of mindfulness-based stress reduction on distressed (Type D) personality traits: a randomized controlled trial

**DOI:** 10.1007/s10865-012-9431-3

**Published:** 2012-05-15

**Authors:** Ivan Nyklíček, Sylvia van Beugen, Johan Denollet

**Affiliations:** 1 CoRPS, Center of Research on Psychology in Somatic disease, Department of Medical Psychology, Tilburg University, Postbox 90153, 5000 LE Tilburg, The Netherlands; 2 Department of Medical Psychology, St. Radboud University Medical Center, Nijmegen, The Netherlands

**Keywords:** Mindfulness, Distressed (Type D) personality, Social inhibition, Negative affectivity, Randomized controlled trial

## Abstract

Distressed (‘Type D’) personality, the combination of negative affectivity (NA) and social inhibition (SI), has been associated with adverse health outcomes. The purpose of this study was to examine if an 8-week mindfulness-based stress reduction (MBSR) program could reduce Type D personality characteristics. Distressed individuals from the Dutch general population (*N* = 146; mean age = 46.07; 69 % female) participated in a randomized trial comparing the mindfulness intervention with waitlist control. Although change in Type D caseness did not differ between groups, the intervention group showed stronger reductions for both NA (*p* < .001) and SI (*p* < .05) dimensions, even when change in state negative affect was statistically controlled. These effects were mediated by change in self-reported mindfulness. In conclusion, MBSR may reduce characteristics of the distressed personality type, likely through the mechanism of increased mindfulness.

## Introduction

The “distressed”, or Type D, personality is defined as the combination of two basic traits: negative affectivity (NA) and social inhibition (SI). NA is the tendency to experience negative emotions across time and situations, which is strongly related to the construct of neuroticism (*r* = 0.64–0.68) (Kupper & Denollet, 2007). SI is conceptualized as the tendency to inhibit the expression of emotions and behaviors in social interactions, which is related to the construct of introversion (*r* = 0.59–0.65) (Kupper & Denollet, 2007). A score higher or equal to 10 on both of the two self-report subscales measuring these dimensions defines Type D caseness (Denollet, 2005). It is a non-psychopathological personality construct that is highly prevalent, with a rate of 13–32.5 % in the general population, 27–31 % in cardiac patients, and up to 45 % in heart failure patients (Denollet, 2005; Pedersen & Denollet, 2006).

The construct of Type D has originally been described and developed in cardiovascular patient groups, and has shown to be an independent predictor of poor health status, increased risk of mortality and increased risk of myocardial infarction in these groups (Denollet et al., 1996; Martens et al., 2010). Type D is also related to psychological problems like decreased quality of life and increased risk of anxiety and depression (Pedersen & Denollet, 2006). Although Type D has mainly been studied in cardiovascular patients, evidence is now emerging that Type D is also a vulnerability factor for decreased physical and mental health status and poor self-management in a wide variety of noncardiovascular patient populations (Mols & Denollet, 2010).

Considering the high prevalence of Type D personality and the associated health risks, it is highly important to explore possibilities for a psychological intervention for patients with this profile. To date, no attention has been paid to the development of interventions that might target Type D personality. Only two studies have been published on the effects of cardiac rehabilitation that included a measure of Type D personality, showing that only the SI component of Type D decreased over the course of group cardiac rehabilitation, while Type D caseness remained stable in 81 % of the participants in both studies (Karlsson et al., 2007; Pelle et al., 2008). In one study, a small reduction in Type D caseness was found after rehabilitation (from 26.6 to 20.7 % cases) (Pelle et al., 2008).

Therefore, in the present study we examined the potential effects of a psychological intervention on the Type D personality traits. The 8-week group mindfulness-based stress reduction intervention (Kabat-Zinn, 1990) was used. It is designed to enhance one’s degree of mindfulness, which is often defined as the state of being attentive to and aware of what is taking place in the present, in an open, accepting, and nonjudgmental way (Brown & Ryan, 2003).

Studies have found positive effects of mindfulness-based stress reduction on reducing general distress and enhancing quality of life (e.g., Nyklíček & Kuijpers, 2008; Speca et al., 2000) and reducing symptoms of anxiety (Miller et al., 1995; Shapiro et al., 1998; Speca et al., 2000) in a variety of patient and healthy populations. A recent meta-analysis showed that mindfulness-based interventions are effective in reducing symptoms of anxiety and depression across a wide range of patient samples (Hedges’ *g* = 0.63 and 0.59, respectively) (Hofmann et al., 2010).

Trait NA and SI may be hypothesized to be also influenced by mindfulness-based interventions. First, beyond the effects found on state negative affect and mood, mindfulness is believed to produce fundamental changes in a person’s appraisal and belief systems (Kabat-Zinn, 1990), which might bring about effects that go beyond momentary mood states and which might influence trait NA as well. Second, SI may also be affected. The general mindful approach of being open and accepting includes viewing oneself with openness and acceptance (Kabat-Zinn, 1990): participants are taught that it is perfectly alright to think, feel, and behave oneself the way one does. This may decrease feeling uncomfortable when finding oneself and expressing oneself in social situations. The group format in which experiences are shared in the same spirit of openness and acceptance might aid in this process.

Therefore, we examined if the standard 8-week mindfulness-based stress reduction intervention could significantly reduce the NA and SI characteristics of Type D personality, even when controlling for change in state negative affect. As the hypothesized changes are expected to be due to changes in mindfulness, it was expected that the effects of the intervention on NA and SI would be mediated by changes in mindfulness.

## Methods

### Participants

The study consisted of two substudies with largely identical, but at some points slightly different procedures. In the first substudy, conducted between August 2005–August 2006, the mindfulness-based stress reduction intervention (*N* = 30) and a waitlist control condition (*N* = 29) were compared regarding the effectiveness on psychological well-being (Nyklíček & Kuijpers, 2008). In the second substudy, conducted between September 2006–January 2009, the intervention (*N* = 44) was compared to a waitlist control group (*N* = 44) regarding mainly effects on physiological stress reactivity in the laboratory (manuscript in preparation).

In both substudies, participants were recruited among community residents by means of advertisements in local newspapers around the city of Tilburg, Netherlands, between August 2005 and October 2007. In these advertisements, people having stress-related complaints were asked to participate in a stress reduction program. Potential participants were asked the following question to verify if they had symptoms of distress: *“how often would you say you feel distressed?”* If their answer could be categorized as *“regularly”* or *“often”,* and exclusion criteria were not met, they were able to participate, as described previously (Nyklíček & Kuijpers, 2008). Exclusion criteria were insufficient understanding of the Dutch language and serious psychopathology (e.g., suicidal ideation or history of psychoticism). All 147 people who applied for participation complied with the inclusion and exclusion criteria and were subsequently randomized. Signed informed consent was provided. The study was conducted according to the Helsinki Declaration of 1975, as revised in 2000, and approved by the Medical Ethics Committee of St. Elisabeth Hospital, located in Tilburg, The Netherlands.

The power analysis was based upon a previous meta-analysis on the effects of mindfulness-based interventions on psychological well-being variables in randomized trials (Grossman et al., 2004), showing a mean medium sized effect size (*d* = 0.5). With an alpha level of 0.05 and a power of 0.80, 63 participants per group were needed for the time by group interaction effect. Taking into account an attrition rate of 10 %, at least 70 participants per group were needed.

### Design

The study was a randomized controlled trial using two parallel groups formed by balanced randomization (1:1). Participants were randomized into either the intervention group, or a waitlist control group. Random selection without stratification was performed using SPSS software (procedure Select Cases) on numbers representing potential participants. The performer of this procedure (first author) received a list with numbers from the second author and did not know which number represented which participant. After randomization, no blinding to group assignment was possible, except for assessment of the outcomes, which was done by sending questionnaires to all participants by post by the second author, who also assigned participants to intervention arms.

### Measures

#### Demographic data

Sociodemographic and basic medical information was obtained regarding age, sex, level of education, job status, and psychotropic medication use.

#### Type D personality

The two Type D personality dimensions were the primary outcome in this study. They were assessed by the Type D Scale-14, in which participants rate their personality on a 5-point Likert scale (Denollet, 2005). The scale consists of seven items which assess NA and seven items which asses SI. Participants are asked to rate to what degree statements are true for them, on a scale ranging from 0 (false) to 4 (true). The questionnaire has shown good reliability (Chronbach’s α’s ranging from 0.86 to 0.88), convergent, discriminant, and predictive validity. A cutoff score of 10 on both the NA and the SI scale is used to classify participants as having a Type D personality (Denollet, 2005).

#### State negative affect

State negative affect was measured using the Positive and Negative Affect Schedule (PANAS) (Watson et al., 1988), except for participants in the first study, who completed the Global Mood Scale (Denollet, 1993). The negative affect subscales of both questionnaires correlate 0.56 (Denollet & De Vries, 2006), making the pooling of their standardized scores possible. The switch to PANAS in the second study was done to examine if the favorable effects found on Global Mood Scale reported previously (Nyklíček & Kuijpers, 2008) also hold for the more frequently used PANAS (the comparison not reported here). Both measures consist of 20 items, of which 10 measure positive affect and 10 measure negative affect. Items are affective words like ‘interested’ or ‘afraid’. Participants are asked to indicate to what extent they have felt that way lately on Likert scales ranging from 1 (very slightly or not at all) to 5 (extremely). Both scales are highly internally consistent (Chronbach’s α’s ranging from 0.84 to 0.90 for PANAS and >0.90 for the Global Mood Scale), while evidence for convergent and discriminant validity has also been provided (Denollet, 1993; Watson et al., 1988).

#### Mindfulness

Mindfulness was assessed using a combination of the Mindful Attention Awareness Scale (Brown & Ryan, 2003) and two subscales of the Kentucky Inventory of Mindfulness Skills (Baer et al., 2004): Observe and Accept Without Judgment. The Mindful Attention Awareness Scale is a 15-item scale designed to measure the frequency of general mindful states in day-to-day life. Respondents can indicate on a 6-point Likert scale how often they experience each condition, ranging from 1 (almost always) to 6 (almost never). Adequate reliability (Cronbach’s α > 0.80), test–retest reliability, discriminant validity and convergent validity have been reported (Brown & Ryan, 2003).

The Kentucky Inventory of Mindfulness Skills is a 39-item mindfulness questionnaire, divided into four scales reflecting four components of mindfulness, of which two were used in the present study: Observe (12 items), and Accept Without Judgment (9 items). Observe refers to noticing whatever happens in the present moment, including mainly sensory sensations (exteroceptive and proprioceptive). Accept without Judgment refers to being non-judgmental and non-evaluative about one’s thoughts and feelings. Items are scored on 5-point Likert scales ranging from 1 (never/rarely true) to 5 (very often/always true). Both subscales have been found to have good reliability (Cronbach’s α of 0.91 and 0.87, respectively), and adequate test–retest reliability and content validity (Baer et al., 2004). The remaining two scales, Describe and Act with Awareness, were not used in the current study. The Act with Awareness subscale has large content overlap with items of the Mindful Attention Awareness Scale, and the Describe subscale refers to descriptions of emotions and feelings, which is not a primary focus of the intervention.

#### Daily practice

Participants who received the intervention were asked at each intervention session how many times in the past week they had practiced mindfulness exercises at home according to the instructions.

### Intervention and procedure

At the pre-treatment assessment, participants were sent the set of questionnaires to fill out at home. After randomization, the intervention group received the 8-week intervention at a meditation center in Tilburg, following the standard mindfulness-based stress reduction protocol (Kabat-Zinn, 1990) as described in detail elsewhere (Nyklíček & Kuijpers, 2008). Briefly, participants followed eight 150 min. weekly group sessions (13–15 participants per group) and a 6-h Sunday retreat in the 6th week of the intervention. During the sessions, besides brief psychoeducation, a combination of mindfulness exercises was taught: breathing and observing mindfully, observing sensations in the body, moving mindfully (adapted hatha yoga exercises) and various forms of sitting mindfulness meditation*.* Participants were expected to practice mindfulness exercises daily at home for at least 40 min.

After 8 weeks the intervention was completed and all study participants were asked again to fill out the questionnaire sets, again per mail, to be filled at home and returned in a postage-free envelope. Hereafter, the waitlist control group also received the intervention.

### Statistical analysis

Data were analyzed using the IBM Statistical Package for the Social Sciences (SPSS), version 19. Before any analyses were done to evaluate the effects of the intervention, successful randomization was checked by comparing the intervention group and the control group on baseline characteristics by means of independent samples *t* tests and χ^2^ tests. All variables were checked for normality of the distributions. Analyses were performed according to the conservative intention-to-treat procedure.

To examine the effects on the scores of the two subscales of the DS-14, which were the primary outcome, multivariate analysis of covariance (MANCOVA) was conducted on change scores from pre- to post-treatment on NA and SI comparing the intervention group and the control group. Using change scores has been shown to provide both a reliable and unbiased estimate of true change (Rogosa, 1988). In this analysis baseline values of the outcome variables were included as covariates, because they often are associated with the degree of change. In addition, difference in state negative affect scores between pre- and post-treatment was also included as covariate, to control for changes in momentary mood. Because two different questionnaires on state negative affect were administered to two subsamples, the values were standardized for these analyses. Any potentially confounding continuous variables that the groups were found to differ on at baseline were added as covariates as well.

Potential differences between effects regarding outcome of separate training groups within the intervention condition were not considered as this was not the primary aim of the investigation and all groups were trained by the same trainer. Nevertheless, to rule out the possibility of large inter-group differences, ANCOVA’s were performed within the intervention condition comparing training groups. These analyses revealed no significant differences on change scores of any outcome variable (all *F* < 1.0; *p* > .10).

Bivariate correlations based on all participants were computed between simple change scores of NA, SI and mindfulness between pre- and post-intervention. Based on the approach of Baron and Kenny (1986), the following assumptions were tested in the mediation analyses to examine the potential mediating effects of mindfulness: (a) the independent variable (group) has a significant effect on the outcome variables (NA and SI), (b) the independent variable (group) has a significant effect on the potential mediator (mindfulness scores), and (c) the potential mediator (change in mindfulness) is associated with the outcome variables (change in NA and SI). If these conditions were met, MANCOVAs including change in mindfulness variables as a covariates were used to examine the mediation effect, as applied in an earlier study (Nyklíček & Kuijpers, 2008). In addition, a nonparametric bootstrap procedure for mediation effects with 5,000 resamples (Preacher & Hayes, 2004) was used to test the indirect effects of mediation statistically. This procedure is recommended above standard Sobel testing as the latter is highly sensitive to the frequently occurring violation of normality of the distribution of the product term of indirect effects (Preacher & Hayes, 2004).

## Results

### Participants flow

**Fig. 1:**
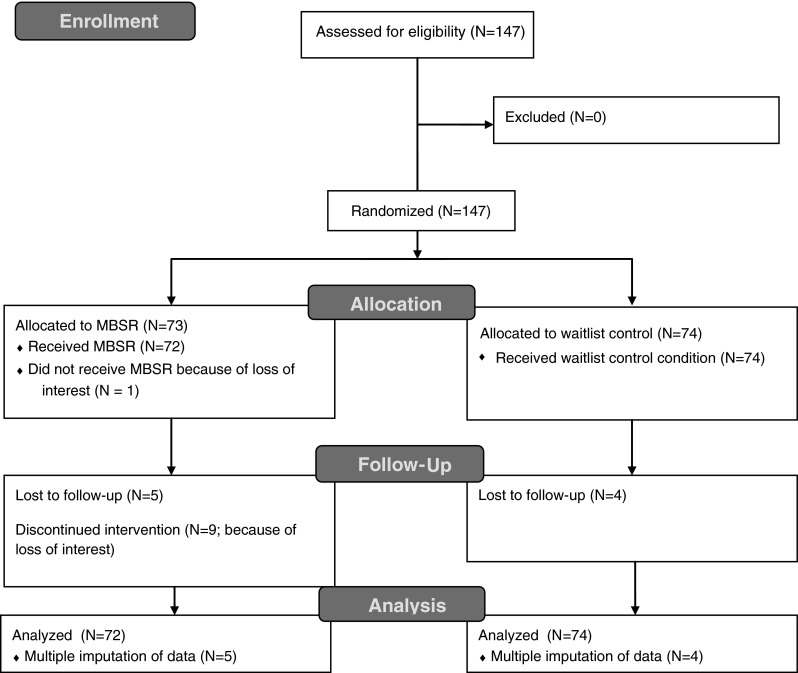
CONSORT flow chart of the participants

Randomization of the 147 individuals resulted in an intervention group of 73 individuals and a control group of 74 persons. One woman who was randomized into the intervention group declined participation before completion of baseline questionnaires due to loss of interest (see Fig. 1 for flow chart).

Nine out of 72 remaining participants (12.5 %) in the treatment group dropped out of the intervention, mainly because of loss of interest. They attended between 0 and 5 sessions (median = 3). Four of these drop outs provided post-intervention data, resulting in five of the 72 participants in the intervention group having missing data at post-intervention regarding outcome and mindfulness variables. In the control group, four of the 74 participants had missing outcome and mindfulness data at post-intervention. A logistic regression analysis predicting missingness was performed in which missing values are recoded as 1 and existing values as 0 and available baseline data are used as predictors. These predictors together explained between 23 % (Cox & Snell *R* ^2^) and 100 % (Nagelkerke *R* ^2^) of the variance of missingness (χ^2^ (13) = 36.27, *p* = .001). The fact that missingness can be predicted by available data may be interpreted as missingness at random, warranting the use of multiple imputation of missing data, based on available data (Graham, 2009; Sterne et al., 2009; Van Buuren & Groothuis-Oudshoorn, 2011).

Occasional missing baseline values were also present. In the intervention group one participant had missing values on education, working status, use of psychotropic medication, and body mass index (BMI) as a result of missing self-reported length. In the control group also one participant had missings on the use of psychotropic medication, BMI, and three participants had missings on NA, SI, state negative affect, and the mindfulness variables. A logistic regression analysis predicting missingness of demographic variables at baseline showed that this could not be significantly predicted by group membership or other baseline variables. In this case, we assume that missing values on demographic variables are unrelated to outcome variables and are, therefore, missings completely at random (Graham, 2009; Sterne et al., 2009). The logistic regression analysis predicting missingness of baseline values of NA and SI showed that group membership, baseline demographic variables, and use of psychotropic medication together predicted between 8 % (Cox & Snell *R* ^2^) and 42 % (Nagelkerke *R* ^2^) of the variance (χ^2^ (4) = 11.55, *p* = .02), suggesting missingness at random that can partially be predicted by other variables.

Therefore, all missing values were imputed using multiple imputation methodology, which is the preferred approach in this case (Graham, 2009; Sterne et al., 2009; Van Buuren & Groothuis-Oudshoorn, 2011). Because some variables are categorical of nature, the Predictive Mean Matching is method is used. Because this method imputes predicted values of a set of individuals with comparable characteristics to those individuals who have missing values on a variable, there is no need to specify an explicit model for the distribution of missing values and its main advantages are: (a) only realistic values are used, (b) it is less vulnerable to model misspecifications (Van Buuren, 2012). Twenty iterations producing 20 imputations were performed using the following variables in the model as it is recommended to use as much of potentially relevant information as available (Sterne et al., 2009): substudy, group, age, sex, education, the amount of working hours per week, BMI, use of psychotropics, dropout, class attendance, home mindfulness practice, state negative affect, NA, SI, Type D categorization, and the three mindfulness variables. To be clear, most of these auxiliary variables are not used in the analyses testing the hypotheses, as stated above. Because the SPSS (M)ANCOVA procedure does not provide pooled estimates for the imputations, the pooled statistics are obtained from equivalent linear regression analyses using the same variables (yielding t-statistics instead of F-statistics). We would like to note that results were the same when analyses were performed based on cases with only complete data (Table 2).

### Baseline characteristics and randomization check

All participants were Caucasian, of which 45 (31 %) were men. Mean age was 46.1 years (SD = 10.3; range 21–66 years). Thirty-nine participants (27 %) had relatively low education (midlevel vocational, high-school or lower), 49 people (34 %) had a job for at least 32 h per week, and 43 individuals (30 %) were on psychotropics (mainly antidepressants). No baseline differences between the groups were found on the demographic variables and Type D personality dimensions (all *p* > .10, Table 1). Baseline prevalence of Type D was much higher in our sample compared to the general population (58 % versus 13–33 %) (Denollet, 2005; Pedersen & Denollet, 2006).

**Table 1:** Baseline characteristics of the sample: original data

	Mindfulness group (*N* = 71–72)	Waitlist control group (*N* = 71–74)	*t* or χ^2^ value
Age	46.7 (11.1)	45.5 (9.6)	−0.74
Female	49 (68 %)	52 (70 %)	0.01
Low education	22 (31 %)	17 (23 %)	0.72
Working ≥ 32 h/week	23 (32 %)	26 (35 %)	0.05
Psychotropics	17 (24 %)	26 (35 %)	1.81
Body mass index	23.9 (4.4)	24.3 (4.0)	0.52
NA	16.0 (5.1)	17.2 (5.00)	1.43
SI	12.4 (6.5)	11.4 (6.3)	−0.93
Type D	41 (57 %)	43 (58 %)	0.00
State negative affect	−0.11 (1.00)	0.11 (1.00)	1.29
General mindfulness	3.44 (0.68)	3.32 (0.68)	−1.06
Accept	31.1 (8.8)	29.2 (7.9)	−1.40
Observe	44.0 (11.6)	44.8 (7.9)	0.45

Low education = high-school, midlevel vocation education or lower; *NA*  negative affectivity, *SI* social inhibition; for state negative affect standardized scores are shown; all *p* > .10

The participants of the two substudies did not differ from each other at baseline on any of the demographic, medical, Type D, state negative affect or mindfulness characteristics (*p* > .10), except for Observe, which was higher in participants of the second study (46.6 ± 10.6) compared to the first study (43.0 ± 9.1); pooled *t* (145) = 2.16, *p* = .03.

### Treatment effects

#### State negative affect

No main effect of time (intercept) was found for state negative affect (pooled *t*(144) < 1, *p* > .10), but a significant group effect appeared (pooled *t*(144) = 3.13, *p* = .002, partial *η* ^*2*^ = 0.05). Inspection of the means showed that only the intervention group decreased from pre- to post-treatment, not the control group (Table 2). Change from baseline to post-treatment correlated with change in trait NA during this period (pooled *r* = 0.36, *p* < .001) and SI (*r* = 0.17, *p* < .05). Therefore, we corrected for change in state negative affect in the analyses on NA and SI. Both uncorrected and corrected results are reported.

**Table 2:** Means (and SD) of Type D dimensions and standardized state negative affect scores at pre- and post-intervention for the mindfulness and control groups

	Mindfulness group (*N* = 72)		Waitlist control group (*N* = 74)		*F/t* values	Effect size partial η^2^
	Pre-treatment	Post-treatment	Pre-treatment	Post-treatment		
NA	16.15 (5.22)	12.99 (5.32)	17.16 (5.09)	16.39 (5.21)	20.83***/4.37***	0.14/0.12
SI	12.49 (6.53)	10.87 (6.45)	11.53 (6.45)	11.17 (6.28)	6.10*/2.54*	0.04/0.04
State negative affect	−0.11 (0.99)	−0.25 (0.97)	0.11 (1.00)	0.28 (0.98)	10.64**/3.13**	0.07/0.06

Data shown are original (non-imputed) data, test statistics are shown for the Time by Group interaction effect: for both non-imputed (*F* test) and imputed data (pooled *t* test), respectively; *NA* negative affectivity; *SI* social inhibition; for state negative affect standardized scores are shown; * *p* < .05; ** *p* < .01; *** *p* < .001

#### Negative affectivity

**Fig. 2:**
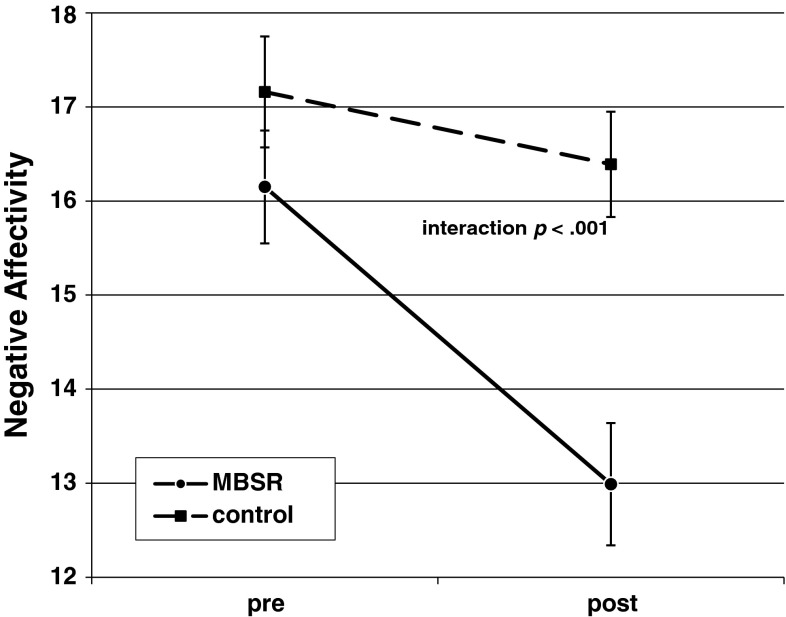
Change in trait negative affect from pre- to post-treatment for mindfulness-based stress reduction (MBSR) and wait-list control group (mean total scores; vertical bars indicate SEMs)

A marginally significant main effect of time (intercept) reflected a tendency for trait NA scores to decrease from baseline to post-treatment across groups (pooled *t*(144) = 1.76, *p* = .08, partial *η* ^*2*^ = 0.02). A significant group effect emerged (pooled *t*(144) = 4.37, *p* < .001, partial *η* ^*2*^ = 0.12): the intervention group showed a larger decrease compared to the control group (Table 2; Fig. 2). Repeating this analysis while controlling for change in state negative affect yielded similar results for the group effect (pooled *t*(144) = 3.69, *p* < .001, partial *η* ^*2*^ = 0.09), while the main effect of time disappeared (pooled *t*(144) < 1.4, *p* > .10). Change in state negative affect showed a significant effect (pooled *t*(144) = 3.65, *p* < .001, partial *η* ^*2*^ = 0.09), as expected.

#### Social inhibition

**Fig. 3:**
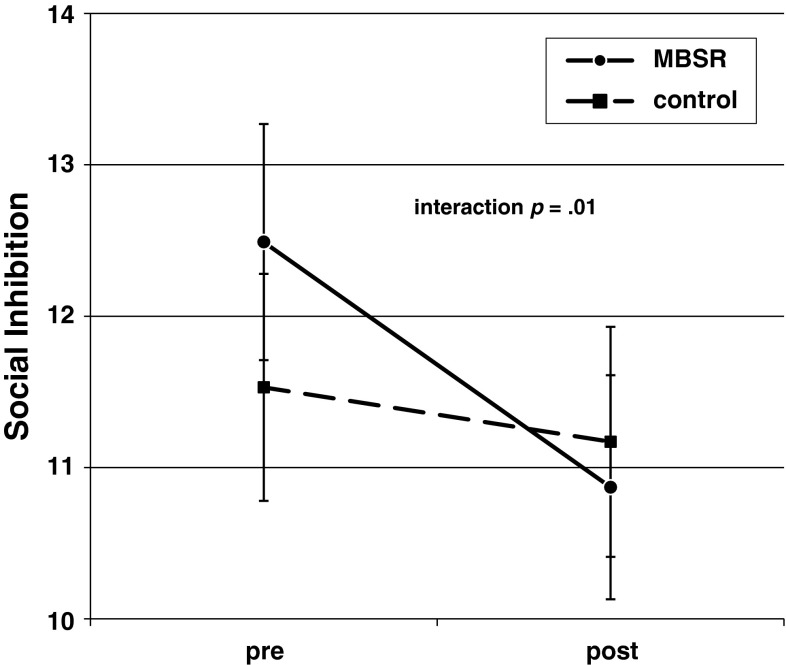
Change in trait social inhibition from pre- to post-treatment for mindfulness-based stress reduction (MBSR) and wait-list control group (mean total scores; vertical bars indicate SEMs)

No main effect of time appeared (pooled *t*(144) < 1.1, *p* > .10), but a significant group effect was present (pooled *t*(144) = 2.54, *p* = .01, partial *η* ^*2*^ = 0.04), showing stronger decrease in the intervention group (Table 2; Fig. 3). Controlling for state negative affect yielded similar results; no main effect of time (pooled *t*(144) < 1.2, *p* > .10), but a significant group effect (pooled *t*(144) = 2.11, *p* = .04, partial *η* ^*2*^ = 0.03). Change in state negative affect showed a marginally significant effect (pooled *t*(144) = 1.93, *p* = .054, partial *η* ^*2*^ = 0.03).

#### Type D caseness

When comparing pre-to post-treatment changes in Type D caseness between the two groups, 9 (to 10 in one imputation) participants out of 41 (22–24 %) Type D individuals at baseline in the intervention group changed to non-Type D post-intervention, compared to 7 out of 43 (16 %) in the control group. Among non-Type D individuals at baseline, 2–4 out of 31 (7–13 %) in the intervention group changed to Type D post-intervention, which was 2–3 out of 31 (7–10 %) in the control group. These changes were not different between groups (*χ* ^*2*^ (2) < 1.59, *p* > 0.45).

#### The role of mindfulness and practice

In an omnibus test of change from pre-to post-intervention of all three mindfulness subscales, a significant main effect of time (intercept: pooled *t*(142) = 4.03, *p* < .001, partial *η* ^*2*^ = 0.26) and a significant effect of group (pooled *t*(142) = 4.25, *p* < .001, partial *η* ^*2*^ = 0.28) emerged. In univariate analyses, the difference between groups was significant for all three mindfulness subscales; for general mindfulness (pooled *t*(144) = 4.57, *p* < .001, partial *η* ^*2*^ = 0.13), Accept Without Judgment (pooled *t*(144) = 2.82, *p* = .005, partial *η* ^*2*^ = 0.06), and Observe (pooled *t*(144) = 5.44, *p* < .001, partial *η* ^*2*^ = 0.17), reflecting a larger increase in mindfulness scores in the intervention group compared to the control group.

In the whole sample, increase in general mindfulness was associated with decreases in NA (pooled *r* = −0.35, *p* < .001), SI (*r* = −0.24, *p* = .004), and state negative affect (*r* = −0.25, *p* = .002) from baseline to post-intervention. An increase in the Accept Without Judgment subscale was associated with a decrease in NA (*r* = −0.31, *p* < .001), but not with changes in SI or state negative affect (*p* > .10), while an increase in the Observe subscale was related to a decrease in SI (*r* = −0.18, *p* = .03), but not with changes in NA or state negative affect (*p* > .10).

Thus, conditions were met for a potential mediation effect by general mindfulness regarding both NA and SI, by Accept Without Judgment regarding NA and by Observe regarding SI. A final ANCOVA analysis was conducted to examine the mediation effects on NA. This analysis was similar to the original analysis on effects on NA, except that in addition to change in state negative affect, pre-post intervention change scores in general mindfulness and Accept Without Judgment were added as covariates. In this analysis, the original group effect was strongly reduced (pooled *t*(142) = 1.97, *p* = .05, partial *η* ^*2*^ = 0.03). The effects of change in general mindfulness (pooled *t*(142) = 1.99, *p* = .047, partial *η* ^*2*^ = 0.03) and change in Accept Without Judgment (pooled *t*(142) = 2.67, *p* = .008, partial *η* ^*2*^ = 0.05) were significant. The bootstrap analyses for mediation effects with 5,000 resamples for both mediators in isolation indicated a significant mediation effect by both general mindfulness (coefficient = 0.63, 95 % CI = 0.18–1.22) and Accept Without Judgment (coefficient = 0.44, 95 % Confidence Interval = 0.02–1.00).

In a similar ANCOVA analysis examining the mediation effects on SI, change in general mindfulness and change in Observe were introduced as covariates. The original group effect was reduced to nonsignificance (pooled *t*(142) = 1.36, *p* > .10). However, the effect of change in Observe was not significant either (pooled *t*(142) = 0.97, *p* > .10), while the effect of general mindfulness approached significance (pooled *t*(142) = 1.78, *p* = .075, partial *η* ^*2*^ = 0.02). The bootstrap analyses for mediation effects for both mediators in isolation indicated a significant mediation effect by change in general mindfulness (coefficient = 0.33, 95 % Confidence Interval = 0.02–0.72) but not by change in Observe (coefficient = 0.25, 95 % CI = −0.15–0.74).

#### Formal home practice

The treatment group practiced on average 4.70 (SD = 1.48) times a week during the entire 8-week intervention period. No associations were found between amount of weekly formal home practice and changes in NA, SI or state negative affect. Of associations with baseline values, only an inverse association was found between weekly home practice and baseline NA (*r* = −0.40, *p* = .001). Regarding session attendance, no associations were found between the number of sessions a participant attended (mean = 4.54; SD = 1.45) and change in NA, SI or state NA.

## Discussion

Current clinical practice lacks a psychological intervention to target characteristics of Type D personality, which is known to be a risk factor for adverse events and poor quality of life in cardiovascular patients (Denollet, 2005; Denollet et al., 1996; Martens et al., 2010; Pedersen & Denollet, 2006). The present findings show a reduction of the negative affectivity (NA) and social inhibition (SI) dimensions of Type D personality as a result of a mindfulness-based stress reduction intervention in normal, albeit distressed individuals. These effects were found even when changes in state negative affect were controlled. This is the first study to show a reduction in characteristics of Type D personality by means of a psychological intervention and also the first randomized study to report changes in any personality characteristics over the course of a mindfulness-based intervention. To the best of our knowledge, only one previous nonrandomized pilot study comparing a mindfulness-based intervention with cognitive-behavioral stress reduction found beneficial effects of the former intervention on scores of neuroticism (Smith et al., 2008), which correlates substantially with the NA dimension of Type D personality (Kupper & Denollet, 2007). In addition, evidence was obtained for a mediating effect of change in mindfulness on both NA and SI, suggesting that change in mindfulness may be the responsible mechanism.

The intervention had a larger effect on NA than on SI. This difference does not seem to be due to NA being possibly more strongly influenced by momentary mood, because the effects remained essentially the same when state negative affect was controlled. This is an important finding, because it is known that current mood states can both increase and decrease scores on personality questionnaires (Lewis, 2001).

Despite the effects of the intervention on SI and NA, we did not find any effects on changes in Type D caseness. After the intervention, 85 % of participants remained stable in Type D caseness. This percentage is comparable to other studies that examined Type D caseness after cardiac rehabilitation (Karlsson et al., 2007; Pelle et al., 2008). The lack of change in Type D caseness could have been due to the fact that our sample contained a large amount of participants with relatively high scores on the Type D scale. While the intervention caused a significant reduction in the scores of both dimensions, they often still remained above the cut-off point, qualifying participants as having Type D personality despite significant and clinically meaningful reductions in their scores. Future studies may examine whether such above cut-off point reductions are capable of reducing cardiovascular risk in cardiac patients with a Type D personality.

Regarding practice effects, it was found that the amount of practice and session attendance were not associated with changes in NA, SI or state NA. This is in line with the inconsistent findings reported in the literature (Nyklíček & Kuijpers, 2008; Shapiro et al., 1998; Speca et al., 2000), although it must be noted that (1) a ceiling effect may have occurred as most participants practiced rather frequently and (2) we did not assess a more fine-grained measurement of, for example, the number of minutes practiced. A correlation was found between baseline NA and weekly practice, suggesting that participants in the intervention who have a tendency to frequently experience negative emotions are perhaps not as inclined to be diligent about home practice compared to participants low on NA.

Consistent with a previous report (Nyklíček & Kuijpers, 2008), associations between change in mindfulness skills and change in outcomes were found for general mindfulness and accepting nonjudgmentally one’s thoughts and feelings, less so for observing phenomena. This may be due to the fact that the latter component, mainly assessing the observation of exteroceptive stimuli, receives less focus in the intervention than the other two facets. In addition, observing exteroceptive stimuli may be less related to one’s affect compared to other facets of mindfulness, as shown previously (Baer et al., 2004). Our anticipated association between increase in accepting nonjudgmentally and decrease in SI was not established. Perhaps the fact that the accepting subscale focuses on accepting one’s thoughts and feelings rather than overt behavior may provide an explanation.

The mediating effect of mindfulness skills regarding change in negative affectivity found in the present study is consistent with previous studies showing that change in mindfulness levels mediates effects of the intervention on psychological well-being (Bränström et al., 2010; Nyklíček & Kuijpers, 2008; Shapiro et al., 2008), and suggest that mindfulness may indeed be the mechanism by which the intervention exerts its effects. The results of the present study extend previous findings by suggesting that changing mindfulness may not only affect psychological states, but also psychological trait characteristics.

### Limitations

One limitation is the relatively high level of education compared to the general population. In addition, all participants were white, and the majority was female. Although we are not aware of studies showing demographic characteristics to be moderators of the effectiveness of the mindfulness-based stress reduction intervention, one cannot exclude the possibility that generalizability of the results to other populations is limited. Future research should include long-term follow-up data to be able to examine if the effects found are stable over a longer period of time. In addition, the inclusion of other active interventions in addition to a waitlist control would enable researchers to examine if the effects are specific to the current intervention.

In conclusion, we found evidence suggesting that mindfulness-based stress reduction can be effective in reducing characteristics of Type D personality, even when controlling for changes in state negative affect. The effects of the intervention on Type D dimensions seem to be mediated by mindfulness skills increase. Future studies should be conducted in cardiac patients, to see if the beneficial effects can be obtained in such populations as well. If so, studies may be set up to examine if the negative prognostic effects of Type D personality in cardiac patients can be reduced as a result of decreased Type D characteristics.
